# The Application of a Design-Based Research Framework for Simulation-Based Education

**DOI:** 10.7759/cureus.31804

**Published:** 2022-11-22

**Authors:** Beheshta Momand, Masuoda Hamidi, Olivia Sacuevo, Adam Dubrowski

**Affiliations:** 1 Health Sciences, Ontario Tech University, Oshawa, CAN

**Keywords:** medical research council framework, design based research, educational research, clinical researcher, healthcare simulation, frameworks, simulation

## Abstract

In research, the adoption of a framework is essential. It enables researchers to operate with specified parameters and provides structure and assistance with research projects, programs, and technologies. The incorporation of a framework also facilitates the organizing and planning of our research efforts with respect to the breadth and depth of what we want to discover. Frameworks are equally important in research focused on simulation-based education. Simulation-based education is a form of experiential learning that provides participants with the opportunity to acquire or improve real-world-like knowledge and skills in a simulated environment. The Medical Research Council framework, historically developed to guide clinical research, has been proposed as a framework to guide simulation research as well. However, because simulation-based education is positioned at the intersection of clinical and educational sciences, certain questions cannot be addressed using a clinical research framework. Thus, in this paper, we introduce an alternative framework, derived from educational sciences, to be considered and possibly adapted into simulation research. The design-based research (DBR) framework consists of four stages centered on design, testing, evaluation, and reflection. This editorial asserts that the DBR is an excellent framework for projects and programs of research in simulation.

## Editorial

Introduction

In research, frameworks help investigators generalize the various aspects of an observed phenomenon by simply describing it and identifying its limits. Frameworks are defined as a structure that can hold or support a theory of a research study [1]. As researchers, when we validate and challenge theoretical assumptions, it advances new knowledge by simplifying concepts and variables in accordance with the presented definitions. A common analogy used to define a framework is providing a blueprint to a builder so that they can construct a home [1]. For researchers, the blueprint provides the parameters for constructing their research. Therefore, frameworks are considered one of the most important aspects of the research process. Frameworks are also important in simulation-based education (SBE). SBE is a type of experiential learning where participants are tasked with solving complex problems in a controlled environment through replicated “real-life scenarios” [2]. SBE can be used to generate awareness and improve clinical skills and attitudes while protecting patients from unnecessary risks. In recent years, SBE has attracted a lot of attention and is growing rapidly. Therefore, due to the increase in popularity of SBE, it has become critical that health professionals have guidance on how to build effective simulation research programmes that provide evidence and subsequently best practice guidelines to help educators to optimize the benefits of SBE.

The Medical Research Council (MCR) framework was proposed as a potential framework in simulation research [3]. Typically, the MCR framework is used to construct and evaluate complex clinical interventions. Although it has been suggested that simulation is a complex intervention regardless of whether it is delivered as a stand-alone experience or as an argumentation of other educational modalities [3], some research questions are difficult to address following this framework. This is primarily because SBE is positioned at the intersection of clinical and educational sciences.

MRC and design-based research (DBR) explained further

In the context of educational sciences, the MRC framework poses a few limitations. For instance, the evaluation phase precedes the implementation phase [3]. This may be problematic because the implementation phase allows researchers to comprehend the various components required to produce the desired effects or outcomes in the real world, as opposed to the tightly controlled research environment. If the development and validation phases of innovation in SBE take several years, finding that there are factors limiting the implementation process would inevitably require the researchers and developers to go back to the early stages of research. This type of setback is disadvantageous, and as a result, the outcomes may not be as significant. Thus, in this paper, we intend to introduce an alternative framework, derived from educational sciences, to be considered and possibly adapted into simulation research, which is called the DBR framework. The DBR framework is typically used by researchers to improve educational practices and learn through instructional tools that can be tested, evaluated, and reflected upon [4]. The DBR framework contains four phases: design, test, evaluate, and reflect. In advance of these phases, it is essential to identify and explore information to a learning problem in order to implement DBR. This will help gather the necessary information in order to execute the design phase. The design phase utilizes existing research and theories to develop appropriate educational resources that will address the theoretical and hands-on issues that surround a learning problem [4]. Instructional tools can include focus groups, worksheets, or different forms of technology that will aid in the development of learning and understanding. During this phase, stakeholders including educators and simulation technologists can contribute to the design phase using a Delphi approach. This will help us determine what considerations must be made when designing the intervention or programme to make it simpler for participants. The testing phase encompasses real-world exposure to instructional tools through implementation in order to assess their effectiveness and determine if they are feasible enough to enhance learning [4]. This phase may differ from the researcher’s initial expectations and may involve continued revision. During the testing phase, stakeholders including learners, educators, and simulation technologists can evaluate the system and content using tools such as the System Usability Scale and the Michigan Standard Simulation Experience Scale framework for assessing the intervention's content. The evaluation phase determines how well the instructional tools were able to produce successful results and what areas need improving [4]. This phase uses evidence from the learner’s deployed experiences and is subject to scrutiny. During this phase, a focus group interview with stakeholders (e.g., learners, educators, and simulation technologists) would be conducted to determine which aspects of the intervention were successful and which ones require improvement. The final phase, known as the reflection phase, involves retrospective examinations of the methodology used to determine if the desired goals were met [4]. During this phase of the framework, Triple Loop Learning can be utilized during a focus group interview with stakeholders to comprehend and evaluate the established design principles and the process.

Benefits of using DBR

When DBR is used, researchers and other stakeholders, such as educators and learners, are all involved at every stage of the process [4]. The objective is for them to interact with programmes, activities, or simulation technologies to improve the learning process and outcomes. This is exceptionally important in simulation research because learners can test their knowledge and learn new ways to solve complex problems. The DBR framework also acknowledges the importance of the evaluation phase, which is a common phrase between the DBR and the MRC. As the DBR testing phase progresses, data collection will be continuously carried out, and new designs and revisions will be implemented to determine what tools best improve learning [4]. Next, in the evaluation phase, the researchers analyze how learners adapt and use educational resources to enhance or hinder their learning and how that information can be used to create a more effective educational tool and programme that promotes learning and understanding rather than impeding it. What is important to our community (i.e., simulation researchers and decision makers) is that the evaluation methodology used in this phase yields the evidence that can inform policies and change in practice. For example, randomized control trials are designed to be the most robust test of effectiveness in the MRC framework, and although not typically used in research framed by the DBR, this design should be considered in the adaptation of DBR to simulation research. Therefore, in summary, there are a series of differences, but also similarities between the MRC and the DBR framework. The main distinction between the two frameworks is seen in the DBR framework's testing and reflecting phases. The testing phase combines the MRC modeling, exploratory trials (i.e., piloting), and implementation phases into a single phase that involves testing the simulation programme or intervention in realistic settings, and this is done very early in the research process. The benefits of incorporating all of this into one phase are that it improves authenticity by testing the programmes in real-world settings, the stakeholders are more engaged in such settings, and feedback generated can be used to improve the technology early in the research and development process, before the formal evaluation and final implementation. The commonalities between these frameworks include the initial theory inform design phases and formal evaluations, although here we would urge the simulation researchers who would like to adapt the DBR to guide their research may want to consider designs, such as randomized control trials, that yield results that are valued by the simulation community most.

How to implement the DBR framework within SBE

In this section, we provide a working example of how DBR can be used to improve nurses’ communication skills when they are conversing with elderly patients who take multiple medications. We highlight the utility of DBR to conduct a programme of research that focuses on the development, modeling, testing, and evaluation of a hypothetical simulation programme and technology that can improve communication skills. In the design stage, the focus is on determining how the programme is constructed, specifically addressing issues of the program’s theory, content, and simulation modalities. In this phase, a focus group interview was held with nurses to understand how to effectively design the intervention. Other techniques, such as design thinking and consensus-building methods (e.g., the Delphi method), can also be used to crowdsource opinions from various stakeholders, such as technologists, researchers, and end-point users. Next, during the testing phase, the programme is implemented in a real-world setting, such as a long-term home, and representatives of the program's target end-point users, in this case, nurses, were recruited to test the programme and technologies and provide feedback on the acceptability, feasibility, and usability. During the evaluation, a cohort and small-scale randomized control trial design involving nurses as participants were used to determine the effectiveness of the programme and supporting educational technologies. Finally, during the reflection phase, a review of the methodology and theories used was conducted. In this step, researchers critically evaluate what had been developed, how it was developed, and whether it was appropriate [3]. 

Conclusion

* *In research, frameworks are used to facilitate the execution of research projects, programmes, and technologies that aim to answer complex research questions. In SBE, frameworks are especially useful in creating effective simulation-based programmes that provide best practices and guidelines to health educators, which is becoming increasingly important as SBE grows in popularity. In the past, the MRC framework was proposed to be utilized in SBE. However, this framework has limitations because simulation-based research frequently focuses on educational issues. The DBR framework, on the other hand, has its roots in the field of education science and offers several novel approaches to designing and implementing research programmes and technologies. One of the most significant contributions of DBR in the interdisciplinary field such as simulation-based education is its focus on early testing of the invention or a programme in an authentic environment. This allows for assessing a potential fit with future implementation context, as well as developing a coherent implementation strategy. Finally, DBR permits reflection on every aspect of the programme in order to inform future research.

This editorial was intended to provide a high-level overview of the DBR framework and compare it to the MRC framework. Intentionally, no concrete tools and protocols are provided, as these will be developed in the future by the simulation research community.
